# The Effect of a Lipopolysaccharide from Rhodobacter capsulatus PG on Inflammation Caused by Various Influenza Strains

**DOI:** 10.32607/20758251-2019-11-3-46-55

**Published:** 2019

**Authors:** S. V. Zubova, M. F. Vorovich, A. S. Gambaryan, A. A. Ishmukhametov, S. V. Grachev, I. R. Prokhorenko

**Affiliations:** Institute of Basic Biological Problems of RAS, FRC PSCBR RAS, Science Ave. 3, Pushchino, Moscow, 142290, Russia; FGBNU Federal Scientific Center of Research and Development of Immunobiological Preparations named M.P. Chumakov of RAS, pos. Institute of Poliomyelitis, Kievskoye Highway, 27th km, 8/1, Moscow Region, 142782, Russia; GAOUVO First Moscow State Medical University named I.M. Sechenov of Russia Health Ministry, Trubetskaya Str. 8, Moscow, 119811, Russia

**Keywords:** influenza virus, H5N1, H1N1, Rhodobacter capsulatus PG lipopolysaccharide, mice, antiviral antibodies, cytokines

## Abstract

The development of a specific inflammation in mice that had been infected by two influenza virus strains, A/chicken/Kurgan/5/2005 (H5N1) and A/Hamburg/2009 MA (H1N1), was studied. We investigated the effect of a non-toxic lipopolysaccharide from Rhodobacter capsulatus PG on the survival and body weight of the mice, production of IgG antibodies, and the induction of pro- and anti-inflammatory cytokines in blood serum. The administration of the R. capsulatus PG lipopolysaccharide was shown to induce interferon-β synthesis, both in healthy and influenza A virus-infected mice, and to promote production of antiviral antibodies in the blood of the influenza-infected animals.

## INTRODUCTION

Influenza epidemics have, to date, affected millions of people across the world despite the use of recommended vaccines, whose effectiveness proves lower than expected [ 1, 2]. Influenza A viruses have a high degree of genomic variation and produce resistant strains that can be controlled by vaccines or antiviral systemic medication for some time. The development of safe and effective vaccines remains an important public health goal.

The interaction of viral components with various receptors activates the intracellular pathways responsible for the secretion of type I IFN, pro-inflammatory cytokines, and chemokines. The key factors involved in the recognition of viral ligands are the Toll-like receptors (TLRs) of innate immune cells. TLR2 and TLR4, situated on the cell surface, recognize viral envelope glyco/lipoproteins, while intracellular endosomal TLR3, TLR7, TLR8, and TLR9 recognize nucleic acids [3, 4]. Toll-like receptors can interact with other receptors, thereby stimulating the response of innate immune cells to pathogens, including influenza viruses [4]. TLR4 can be activated by damage-associated molecular patterns (DAMPs), which are molecular structures released by virus-infected cells [5]. Different influenza strains activate cells through various mechanisms, which lead to the synthesis of various cytokines and chemokines [6, 7].

Compound E5564 (Eritoran), a synthetic analogue of the non-toxic lipid A from Rhodobacter sphaeroides, when administered in a certain regimen to C57BL/6J mice, was shown to protect mice from death caused by the mouse-adapted H1N1 influenza virus [8]. The nuclear non-histone high mobility group box 1 (HMGB1) protein/amphoterin, which is a DAMP, is known to be released relatively late after the infection onset and is involved in the development of both gram-negative sepsis and influenza complications, interacting with MD-2 and activating TLR4 [5, 9, 10]. TLR4 activation leads to a cytokine storm with an accentuated release of pro-inflammatory cytokines, including interferons, tumor necrosis factors, interleukins, and chemokines [11]. Pharmacological blockade of TLR4 by Eritoran can significantly reduce mouse mortality from avian influenza [8]. A lipopolysaccharide (LPS) from a phototrophic bacterium R. capsulatus PG (Rb.) strain [12], with a lipid A structure similar to that of lipid A from R. sphaeroides, is an endotoxin antagonist that inhibits activation of the synthesis of numerous pro-inflammatory cytokines by human blood cells [13], an indication of its ability to block TLR4.

Mice are the main tools used for studying the human immune system and immune responses. However, there are significant differences between the innate and adaptive immune systems of mice and those of humans, which reside in the blood cell ratio, plasma composition, surface receptors, the expression levels of various cytokines and chemokines, etc. [14, 15]. This should be considered when using mice as human disease models.

In this paper, we studied the effect of a non-toxic Rb. LPS on the induction of pro- and anti-inflammatory cytokines and survival rates of mice infected with various influenza A strains. The study aim was to investigate the features of the inflammatory processes caused by H1N1 and H5N1 influenza viruses.

## EXPERIMENTAL

The following ELISA kits were used: mouse TNF alpha platinum ELISA, mouse IL-6 platinum ELISA, mouse IL-10 platinum ELISA, and mouse INF gamma platinum ELISA (eBioscience, USA), as well as a mouse IFN beta ELISA kit (PBL Assay Science, USA).

The Rb. LPS was produced in a laboratory of the Institute of Basic Biological Problems, according to the procedure described previously [16].

**Viruses**

We used the following influenza A virus strains: chicken/Kurgan/5/2005 (H5N1) and mouse-adapted Hamburg/2009 MA (H1N1). Viruses were cultured in chicken embryos. The virus median tissue culture infectious dose (TCID50) was determined by titration in a Madin-Darby canine kidney (MDCK) cell culture. The median lethal dose (LD_50_) was determined by titration in mice. Experiments with the highly pathogenic A/ chicken/Kurgan/5/2005 virus were performed in boxes with the BSL-3 safety level.

**Mice**

We used 10–14 g Balb/c mice, 36–38 days of age, regardless of gender. The animals originated in the nursery of the Scientific Center for Biomedical Technology of the Federal Medical and Biological Agency. All manipulations with the animals were performed according to the Rules of Laboratory Practice in the Russian Federation [17], in compliance with biological ethics in experiments on laboratory animals.

**Experimental influenza infection in mice infected with the influenza virus strain H5N1**

**Fig. 1:**
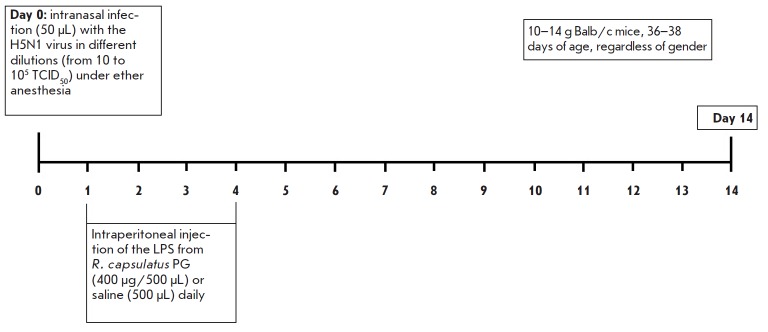
Design of the experiment on the infection of mice with the A/chicken/Kurgan/5/2005 (H5N1) influenza virus strain

Mice were divided into six groups depending on the received drugs. Each group included at least 12 animals. The animals in the five groups were infected, under light ether anesthesia, intranasally (50 μL each) with the highly pathogenic avian influenza virus H5N1 at doses of 10 to 105 TCID50 per mouse, which amounted to 10–1 to 103 LD_50_. The sixth control group remained uninfected. After 24 h, all groups were divided in half. One half of the animals was injected intraperitoneally with 500 μL/mouse of saline daily for the following 4 days, and the other half was injected with the Rb. LPS at a dose of 400 μg/500 μL/mouse. Mice not injected with any agents were the controls. The experimental design is shown in Fig. 1. Blood was sampled from the animals that survived by the end of the experiment (day 14), after euthanasia in a CO_2_ chamber; blood cells were precipitated; and the resulting serum was frozen at –20°C until the H5N1 influenza virus antibody titer in the serum was determined by ELISA.

**Experimental influenza infection in mice infected with the mouse adapted influenza virus strain H1N1**

The mice-adapted pandemic virus H1N1 used in the study was passed serially 20 times in a mouse lungs and differed from the parent A/Hamburg/2009 (H1N1) strain by a deletion in neuraminidase (NA) and mutations in the HA, NP, PA, and PB1 proteins (Table). The H1N1 influenza virus is 105–fold more pathogenic for mice compared to the initial parent strain.

**Table 1:** Substitutions in the A/Hamburg/2009 virus during adaptation to mice

Viral strain	Viral protein, amino acid sequence						
	NA	HA			NP	PA	PB1
	56–67	158	224	225	289	92	317
Hamburg/2009		G	R	D	H	N	M
Hamburg/2009 MA	Del 56-67	E	K	G	Y	S	V

**Fig. 2:**
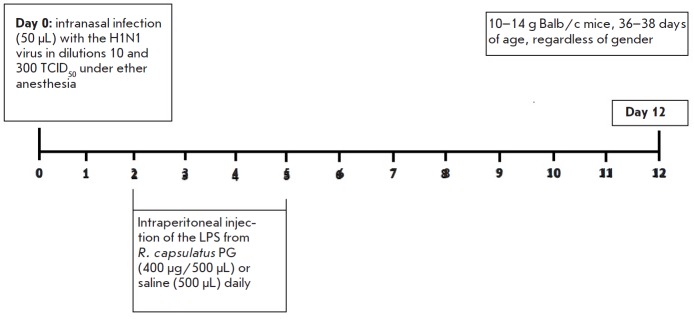
Design of the experiment on the infection of mice with the A/Hamburg/2009 MA (H1N1) influenza virus strain

The mice were divided into three groups depending on the received drugs. Each group included at least 12 animals. Two groups of mice were infected, under mild ether anesthesia, intranasally (50 μL each) with the mouse-adapted H1N1 influenza virus at doses of 10 and 300 TCID50 per mouse. The control group included uninfected mice. Two days after infection, all groups were divided in half. One half of the animals was injected intraperitoneally with 500 μL/mouse of saline daily for the following 4 days, and the other half was injected with the Rb. LPS at a dose of 400 μg/500 μL/mouse. Mice not injected with any agents were the controls. The experimental design is shown in Fig. 2. Five hours after administration of the Rb. LPS, blood was sampled in three mice from each group, after euthanasia in a CO_2_ chamber, on days 3, 4, and 5. The blood was centrifuged, and the resulting serum was frozen at –20 °C until the cytokine levels were determined by ELISA. Blood was sampled from the animals that had survived by the end of the experiment (day 14), after euthanasia in a CO_2_ chamber; blood cells were precipitated, and the resulting serum was frozen at –20 °C until the serum levels of the H1N1 influenza virus IgG1 and IgG2a antibodies were determined by ELISA.

**Determining Cytokine Levels**

Levels of TNF-α, IL-6, IL-10, IFN-γ, and IFN-β cytokines in the blood serum of mice infected with the H1N1 influenza virus were determined using ELISA kits according to the manufacturer’s recommended procedure. The optical density of the samples was measured using a STAT FAX 3200 immunoassay analyzer (Awareness, USA) at a wavelength of 450 nm.

**Determining influenza virus antibody levels**

## EXPERIMENTAL PROCEDURES

To determine the levels of antibodies to hemagglutinin (HA) of the H5N1 and H1N1 influenza viruses in the serum of the mice, allantoic fluid containing 64 HA units of one of the viruses was added to a plate sensitized with fetuin, kept at 40°C overnight, washed with phosphate-buffered saline (PBS) pH 7.4 with 0.1% Tween-20, and blocked with buffer A (0.1% Tween-20, 0.2% BSA in PBS) for 1 h. To determine the IgG1 and IgG2a antibodies, each serum sample was titrated on two different plates. The blocking solution was removed, and wells were added with 100 μL of the serum from H5N1-infected mice at 1 : 40 to 1 : 2,560 dilutions or from H1N1-infected mice at 1 : 50 to 1 : 3,200 dilutions in buffer A. Plates were incubated at 40°C for 4 h, washed with PBS, added with horseradish peroxidase- labeled rabbit antibodies against mouse immunoglobulins (Sigma, USA) or against mouse IgG1 or IgG2a, and incubated at 40°C for 2 h. Then, the plates were washed with PBS and stained with ortho-phenylenediamine. The optical density of the samples was measured using an AIFR-01 Uniplan immunoassay analyzer (Picon, Russia) at a wavelength of 492 nm. Control wells without viral particles were used to exclude nonspecific binding. The antibody level in the samples was expressed as serum dilution enabling a signal exceeding twice the background value.

**Statistical analysis**

Microsoft Office Excel 2010 (AtteStat plugin) and OriginPro 7.5 were used for statistical analysis and graphical presentation of our data. Statistically significant differences between the results were evaluated using a nonparametric Mann–Whitney U-test. Differences were considered significant at a significance level p < 0.05.

## RESULTS AND DISCUSSION

**Fig. 3:**
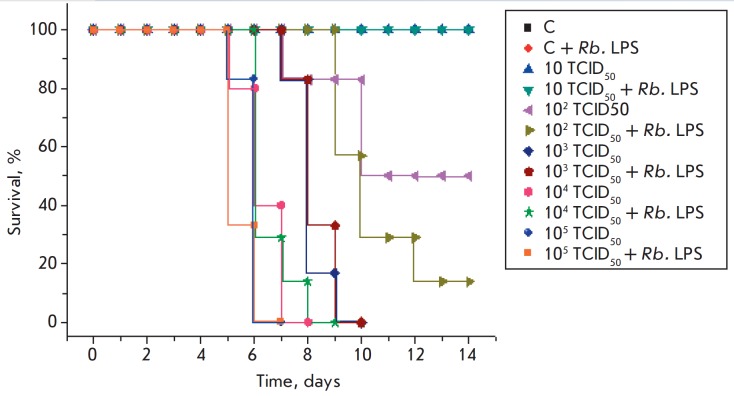
Survival of mice in response to the administration of the Rb. LPS, A/chicken/Kurgan/ 5/2005 (H5N1) influenza virus, and both factors simultaneously

**Fig. 4:**
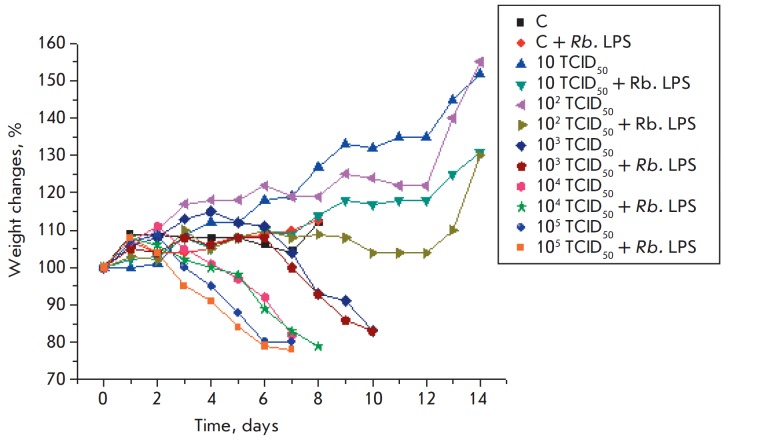
Weight changes in mice in response to the administration of the Rb. LPS, A/chicken/ Kurgan/5/2005 (H5N1) influenza virus, and both factors simultaneously

The condition of the experimental animals was evaluated based on survival and body weight changes. The administration of the Rb. LPS into the control mice, as well as infection with a minimum dose of 10 TCID50 of the H5N1 virus, regardless of Rb. LPS administration, did not affect the survival rates of the animals up to the end of the experiment (day 14) (Fig. 3). Deaths of animals in the groups infected with doses of 102/103 and 104/105TCID50 of the H5N1 virus began on days 8 and 6 after infection, respectively. All mice that had received 103, 104, and 105 TCID50 of the influenza virus died by day 10 after the infection, regardless of Rb. LPS administration. Additional administration of the Rb. LPS to mice infected with the influenza virus at a dose of 102 TCID50 increased their mortality (Fig. 3). The curves of weight changes revealed that introduction of the Rb. LPS into healthy animals did not affect their condition and weight (Fig. 4). Mice infected with 10–102 TCID50 of the virus continued to gain weight throughout the experiment. Infection with doses of 103–105 TCID50 significantly affected the condition of the animals, causing significant inflammation and rapid weight loss. Additional administration of the Rb. LPS to infected animals led to even greater weight loss (Fig. 4).

**Fig. 5:**
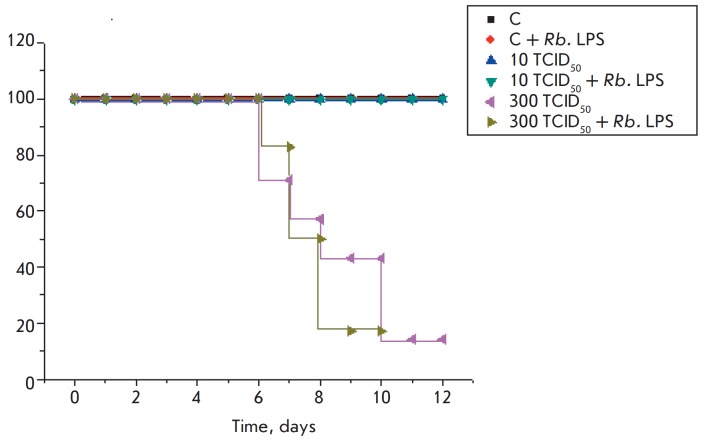
Survival of mice in response to the administration of the Rb. LPS, A/Hamburg/ 2009 MA (H1N1) influenza virus, and both factors simultaneously

**Fig. 6:**
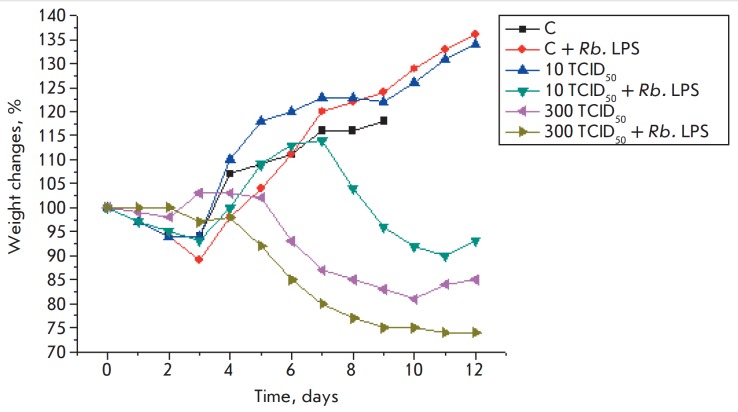
Weight changes in mice in response to the administration of the Rb. LPS, A/ Hamburg/2009 MA (H1N1) influenza virus, and both factors simultaneously

The administration of the Rb. LPS to the control mice and infection with a dose of 10 TCID50 of the H1N1 influenza virus, followed by the administration of the Rb. LPS, did not affect the survival rate of the animals until the end of the experiment (day 12). In the group of mice infected with the influenza virus at a dose of 300 TCID50, 14% of the mice survived until the end of the experiment; additional administration of the Rb. LPS increased mortality in mice (Fig. 5). An analysis of weight change curves showed that the administration of the Rb. LPS to healthy mice did not affect the condition and weight of the control animals. Mice infected with 10 TCID50 of the H1N1 virus showed no signs of disease and began rapidly gaining weight on the 3^rd^ day after infection, until the end of the experiment (day 12). Additional administration of the Rb. LPS to the animals infected with the virus at this dose led to a decrease in the animals’ weight after day 7 of the experiment. Virus infection (300 TCID50) markedly affected the condition of the mice, causing significant inflammation and weight loss. The administration of the Rb. LPS to infected mice worsened the condition of the animals and led to additional weight loss (Fig. 6).

Survival and weight changes in the animals infected with the H5N1 or H1N1 influenza virus revealed the absence of a protective effect on the part of the Rb. LPS on mice against a lethal infection (Fig. 3, Fig. 4, Fig. 5 and Fig. 6). Daily (for 5 days) intravenous administration of Eritoran starting 2 days after infection was shown to protect mice from the A/PR/8/34 (H1N1) influenza virus. Protection from DAMPs released from influenza infected and destroyed cells that occurred through the TLR4- dependent mechanism [18]. The virus type determines the response mechanisms of innate immunity to infection. The signaling pathways in an infection caused by different H5N1 and H1N1 virus strains differ and determine the survival rate and pathology of the inflammation [7]. Obviously, the infection in our experiments develops through molecular mechanisms other than cell activation through the TLR4 pathway. Probably, the administration regiment and Rb. LPS concentration used in our experiments were ineffective in protecting against these viral strains.

Signs of viral infection include an increased induction of pro-inflammatory cytokines and chemokines, such as TNF-α, IL-1, IL-6, IL-8 [19], as well as IFN-β and IFN-γ, which have antiviral effects [20, 21].

**Fig. 7:**
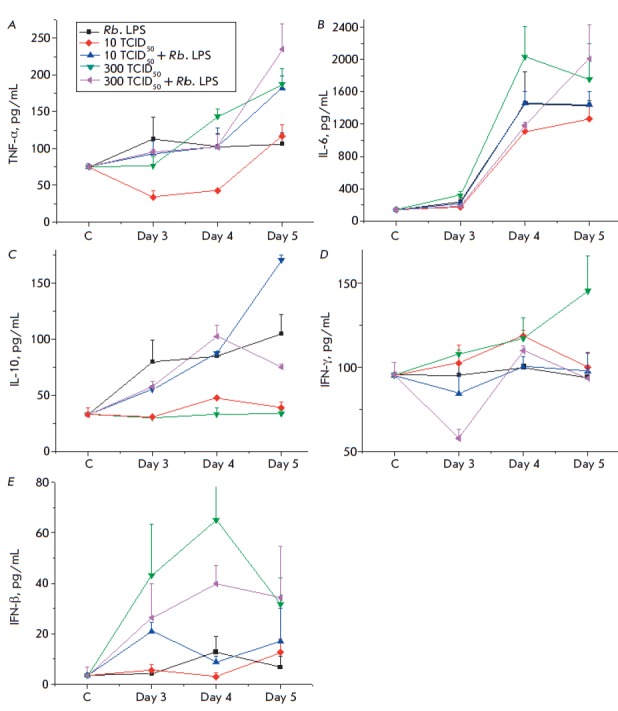
Dynamics of cytokine levels in response to the administration of the Rb. LPS, A/Hamburg/2009 MA (H1N1) influenza virus, and both factors simultaneously. A – TNF-α, B – IL-6, C – IL-10, D – IFN-γ, E – IFN-β

The administration of the Rb. LPS to mice caused a 1.5-fold increase in the TNF-α level in blood serum compared to that in the control mice by day 3, which remained at about the same level until day 5 of the experiment. It should be noted that the serum TNF-α level in the control mice was quite high (74.7 ± 8.7 pg/mL), indicating a sensitized state of the animals. Production of TNF-α in the blood of the influenza-infected mice depended on the virus dose and showed positive dynamics during the experiment. The administration of the Rb. LPS to infected mice enhanced TNF-α production in their blood (Fig. 7A).

The dynamics of IL-6 synthesis in all experiment variants was similar. The IL-6 level increased significantly (1000- to 2000-fold) by day 4 of the experiment. IL-6 production in the infected mice depended on the virus dose. The IL-6 level in response to the introduction of the Rb. LPS was comparable to that in the influenza-infected mice. The administration of the Rb. LPS to infected mice slightly increased IL-6 production by day 5 of the experiment (Fig. 7B). These findings demonstrate that a viral infection causes a dose-dependent induction of the synthesis of the pro-inflammatory cytokines TNF-α and IL-6, with the induction increasing with time. These cytokines are produced mainly by monocytes and macrophages in response to both bacteria and viruses, using independent signaling pathways involving different surface and intracellular receptors but the same adapter proteins and transcription factors. The data on the induction of TNF-α and IL-6 synthesis show that the administration of the Rb. LPS to influenza-infected mice enhanced the pro-inflammatory response of their immune cells (Fig. 7A, B). This worsened the condition of the animals, as evidenced by the data on the survival rate and weight changes (Fig. 5, Fig. 6).

The administration of the Rb. LPS into mice caused an increase in the anti-inflammatory IL-10 cytokine level that exceeded the baseline level 3-fold by day 5. The influenza virus, regardless of the dose, had no effect on the production of the anti-inflammatory IL-10 cytokine. The administration of the Rb. LPS into infected mice, regardless of the virus dose, had almost no bearing on the induction of IL-10 synthesis compared to the administration of the Rb. LPS alone (Fig. 7C). The immunoregulatory cytokine IL-10 is a key component of the system that regulates excessive immune responses by suppressing the expression of pro-inflammatory cytokines such as TNF-α, IL-6, and IL-1 [22, 23]. Levels of IL-10 production in response to LPS are significantly higher than those in a viral infection [24, 25]. The obtained results indicate an increase in the IL-10 level in response to the administration of the Rb. LPS into both healthy and influenza-infected mice. This may indicate that the Rb. LPS promotes enhanced anti-inflammatory responses by cells (Fig. 7C).

These findings indicate a lack of IFN-γ production in response to the administration of the Rb. LPS to mice. The influenza virus increases the blood IFN-γ level in mice in a dose-dependent manner. Furthermore, additional administration of the Rb. LPS to mice infected with the H1N1 influenza virus reduced IFN-γ production in their blood (Fig. 7D).

Having no effect on the production of IFN-γ, the Rb. LPS caused a 4-fold increase in the IFN-β production compared to the control level by day 4 of the experiment. On day 5, the IFN-β level had decreased to its baseline value. In response to a H1N1 virus infection at a dose of 10 TCID50, the IFN-β level increased 3-fold by day 5 of the experiment. The influenza virus at a dose of 300 TCID50 significantly increased the induction of IFN-β (16-fold), compared to the baseline value by day 4. The administration of the Rb. LPS to mice infected with 10 TCID50 and 300 TCID50 of the influenza virus enhanced or decreased, respectively, the cytokine level, compared to that in mice infected with the influenza virus alone (Fig. 7E).

These results demonstrated that the levels of IFN-γ and IFN-β synthesized in response to the used Rb. LPS administration schedule were insufficient for an effective antiviral protection against the virus strains under study.

IFN-β, a component of the influenza vaccine, acts as a powerful adjuvant and helps induce the synthesis of IgG2a and IgA, providing protection against infection. Production of IgG2a antibodies, which is characteristic of the response to a viral infection, has a protective and neutralizing effect against influenza viruses. Expression of type I IFN and generation of IgG2a antibodies in a viral infection are interrelated events of biological significance for subsequent protective immunity [26].

**Fig. 8:**
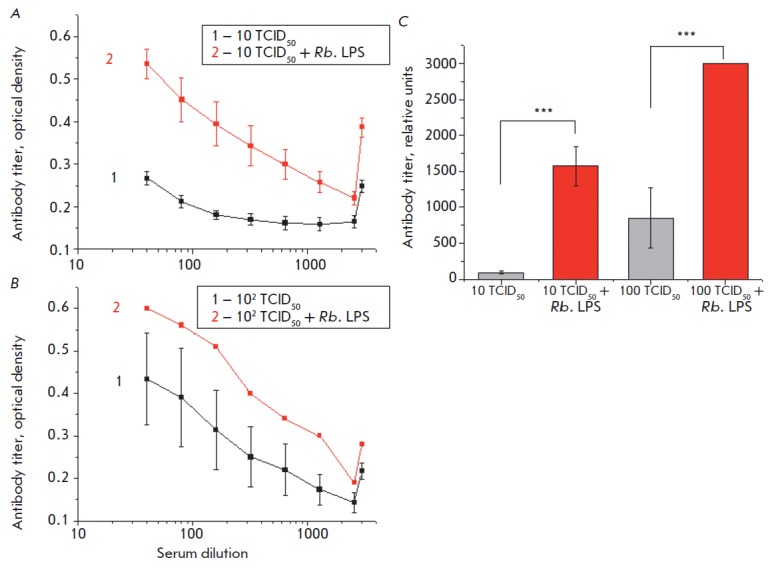
Effects of the Rb. LPS on the levels of antibodies to the A/chicken/Kurgan/5/2005 (H5N1) influenza virus in the serum of mice survived by day 14 of the experiment. A – virus dose of 10 TCID50; B – virus dose of 102 TCID50; C – antibody titer in relative units. ***p < 0.001

The action of existing vaccines against an influenza virus infection is based mainly on the induction of neutralizing antibody synthesis in response to viral HA [27]. Determination of the level of H5N1 virus HA antibodies in mouse serum revealed that the higher the infective virus dose, the higher the titer of serum antibodies in the infected animals. Additional administration of the Rb. LPS to H5N1-infected mice resulted in a significant increase in the antibody titer (p < 0.001) in blood serum (Fig. 8).

**Fig. 9:**
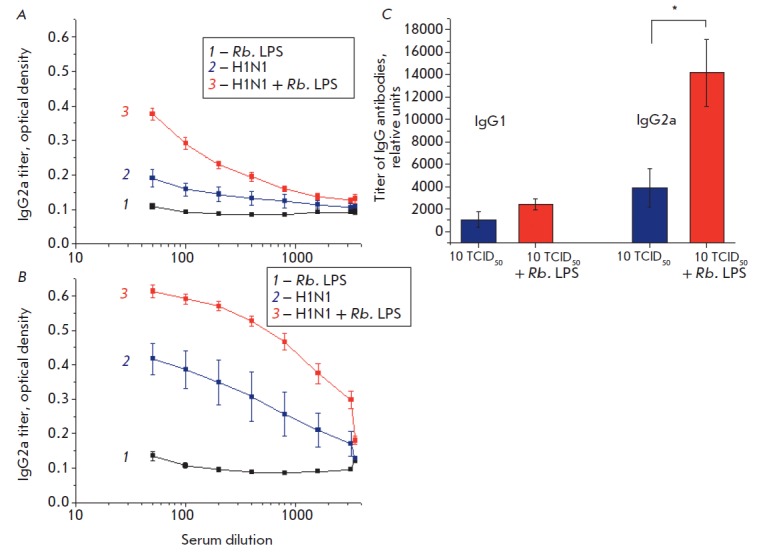
Effects of the Rb. LPS on the levels: A – IgG1 and B – IgG2a antibodies to the A/Hamburg/2009 MA (H1N1) influenza virus (virus dose of 10 TCID50) in the serum of mice survived by day 12 of the experiment. C – titer of IgG antibodies in relative units. *p < 0.05

Determination of IgG1 and IgG2a antibody levels in response to the H1N1 virus infection showed that IgG2a titers were significantly higher than the IgG1 titers in all the groups of animals. Introduction of the Rb. LPS statistically significantly increased the serum IgG2a level compared to influenza-infected mice without additional administration of the Rb. LPS (p < 0.05) (Fig. 9).

The innate immune response is crucial in the fight against viruses and plays a key role in the induction and regulation of adaptive immune responses. For this reason, TLR ligands are considered as potential adjuvants for inclusion in vaccines. Simultaneous delivery of a TLR ligand and an antigen of interest is believed to be more effective than vaccination with a mixture of an adjuvant and an antigen [28, 29].

Our findings demonstrate that the administration of the Rb. LPS to mice promotes IFN-β production (Fig. 7E) or controls the blood IFN-β level in mice infected with various doses of the influenza virus. IFN-β promotes antibody production by acquired immunity cells [26]. Our findings also demonstrate that additional administration of the Rb. LPS leads to the production of antibodies in the blood of animals infected with the influenza A virus (Fig. 8, Fig. 9).

## CONCLUSION

Our study has demonstrated that the nontoxic natural Rb. LPS promotes the production of the immunomodulatory cytokine IFN-β both in healthy mice and in animals infected with influenza A/chicken/Kurgan/ 5/2005 (H5N1) and A/Hamburg/2009 MA (H1N1) strains and also promotes the production of antibodies to the HA of these strains in the blood of infected animals.

## Abbreviations

DAMP: damage-associated molecular pattern
HA: hemagglutinin
HMGB1: high mobility group box 1 protein/amphoterin
IFN: interferon
Ig: immunoglobulin
IL: interleukin
MD-2: myeloid differentiation protein 2
MDCK cells: Madin-Darby canine kidney cells
MyD88: myeloid differentiation protein 88
NP: nucleoprotein
PA, PB1: polymerase complex proteins
TLR: Toll-like receptor
TNF: tumor necrosis factor
TRAF6: TNF receptor-associated factor 6
ELISA: enzyme-linked immunosorbent assay
LPS: lipopolysaccharide
